# Random forests on Hadoop for genome-wide association studies of multivariate neuroimaging phenotypes

**DOI:** 10.1186/1471-2105-14-S16-S6

**Published:** 2013-10-22

**Authors:** Yue Wang, Wilson Goh, Limsoon Wong, Giovanni Montana, the Alzheimer's Disease Neuroimaging Initiative

**Affiliations:** 1 Graduate School for Integrative Sciences and Engineering, National University of Singapore, Singapore; 2 School of Computing, National University of Singapore, Singapore; 3 Department of Computing, Imperial College London, UK; 4 Department of Mathematics, Imperial College London, UK

## Abstract

**Motivation:**

Multivariate quantitative traits arise naturally in recent neuroimaging genetics studies, in which both structural and functional variability of the human brain is measured non-invasively through techniques such as magnetic resonance imaging (MRI). There is growing interest in detecting genetic variants associated with such multivariate traits, especially in genome-wide studies. Random forests (RFs) classifiers, which are ensembles of decision trees, are amongst the best performing machine learning algorithms and have been successfully employed for the prioritisation of genetic variants in case-control studies. RFs can also be applied to produce gene rankings in association studies with multivariate quantitative traits, and to estimate genetic similarities measures that are predictive of the trait. However, in studies involving hundreds of thousands of SNPs and high-dimensional traits, a very large ensemble of trees must be inferred from the data in order to obtain reliable rankings, which makes the application of these algorithms computationally prohibitive.

**Results:**

We have developed a parallel version of the RF algorithm for regression and genetic similarity learning tasks in large-scale population genetic association studies involving multivariate traits, called PaRFR (Parallel Random Forest Regression). Our implementation takes advantage of the MapReduce programming model and is deployed on Hadoop, an open-source software framework that supports data-intensive distributed applications. Notable speed-ups are obtained by introducing a distance-based criterion for node splitting in the tree estimation process. PaRFR has been applied to a genome-wide association study on Alzheimer's disease (AD) in which the quantitative trait consists of a high-dimensional neuroimaging phenotype describing longitudinal changes in the human brain structure. PaRFR provides a ranking of SNPs associated to this trait, and produces pair-wise measures of genetic proximity that can be directly compared to pair-wise measures of phenotypic proximity. Several known AD-related variants have been identified, including APOE4 and TOMM40. We also present experimental evidence supporting the hypothesis of a linear relationship between the number of top-ranked mutated states, or frequent mutation patterns, and an indicator of disease severity.

**Availability:**

The Java codes are freely available at http://www2.imperial.ac.uk/~gmontana.

## Introduction

The last few years have seen extensive efforts to correlate human genetic and phenotypic variation. An increasing number of population genome-wide association studies (GWAS) have been carried out to discover causal associations between common genetic variations and complex human traits. These studies rely on high-throughput platforms that measure genetic changes at hundreds of thousands or even million single-nucleotide polymorphisms (SNPs) across the human genome in large random samples. Full sequencing of human genomes has shown that, in any given individual, there are on average approximately 4 million genetic variants [1]. The most common study design generally involves comparing a sample of healthy control subjects with a sample of diseased subjects, with the goal of identifying patterns of polymorphisms that vary systematically between these two populations and could, therefore, represent the effects of risk-enhancing alleles. Such abundance of genetic markers has now made it possible to identify quantitative trait loci (QTL), which are regions of a chromosome or even individual sequence variants that are responsible for trait variation. For many diseases, such as asthma or attention deficit hyperactivity disorder (ADHD), investigators routinely measure multiple *endophenotypes *that are thought to be more proximal to the biological etiology of the clinical disorder [2].

Traditional statistical genetics methodologies have started to be complemented with, or even replaced by, machine learning algorithms because they often make minimal assumptions about the underlying causal disease mechanism, which is generally unknown. Case-control studies can be analysed by performing SNP selection and ranking in the context of pattern classification. Random Forest (RF), which is amongst the top performing algorithms for supervised learning, has been found particularly promising in case-control studies [3-6]. Phenotypic variation in human populations is typically due to underlying genetic complexity from multiple interacting loci, with allelic effects that are sensitive to the environmental conditions each individual experiences. RF has also been regarded as a particularly powerful approach to capture gene-environment interactions and epistatic effects [7-10].

Our interest in this work is on detecting genetic variants associated to quantitative, and possibly multivariate and very high-dimensional, traits. When a QTL is found to be linked to a causative marker locus, then individuals with different marker locus genotypes will have different mean values of the quantitative trait. In this respect, the QTL mapping problem can also be treated as a feature selection and ranking problem, albeit in a regression setting. Several studies mapping QTL that affect human diseases and complex traits have uncovered new loci. Although much emphasis has been placed on linkage mapping, or QTL mapping in families, there is now increasing interest for QTL mapping in unrelated individuals from the same population, or association mapping [11].

An instance of association mapping with very high-dimensional quantitative traits is found in the area of *neuroimaging genetics*, an emerging field that is rapidly identifying genetic variants that influence the brain structure and function [12-14]. Many research groups are now scanning unrelated individuals with structural and functional MRI (Magnetic Resonance Imaging), DTI (Diffusion Tensor Imaging) and other imaging modalities to characterise variability in the brain. In whole-brain studies, an imaging phenotype may consist of thousands or millions of measurements in a 3D space, representing for instance gray matter intensities. These studies create important statistical challenges due to the very high dimensionality of the quantitative trait being observed. Power gains can be expected by analysing all these measurements jointly, rather than performing multiple independent analyses each involving a univariate response or using summary measures [15]. The use of such multivariate heritable imaging signatures of disease may increase the power to detect causal variants, when compared with a simpler case-control status, since gene effects are expected to be more penetrant at the imaging level, rather than at the diagnostic level [16,17]. Although neuroimaging genetics studies have already identified coherent anatomical patterns of gene effects in three-dimensions using advanced statistical methods [15,18-20], the potential of machine learning methods in that area has not yet been fully explored, and this may be due to the lack of scalable implementations.

We describe here a parallel implementation of RF for regression problems with multivariate responses which allows to quantify the importance of each genetic marker in predicting the trait. Our implementation has been specifically designed to run on large Hadoop clusters, including those available through cloud computing services such as Amazon Elastic MapReduce. The Hadoop ecosystem consists of a set of tools for building distributed systems, including tools for storage, data analysis, and coordination, thus enabling algorithms to be run on thousands of computational nodes. Hadoop was originally designed to address two main issues that arise when distributing data and computations across a very large cluster. First, the problem of hardware failure, which is addressed through replication; redundant copies of the data are kept by the system so that in the event of failure, there is always another copy available. Second, the problem of reliably combining the data resulting from various parallel computations from potentially many nodes and disks. The latter problem is addressed by adopting the MapReduce programming model [21]. Programs written in this functional style are parallelized and executed on a large cluster of commodity machines. Hadoop is currently an open source Apache project.

The paper is structured as follows. Section Methods provides a description of the RF algorithm with multivariate responses, including an alternative node splitting criterion for tree building that is computationally convenient when the trait is high-dimensional, and a procedure for ranking SNPs in order of their predictive importance. We also present the strategy adopted to parallelize the algorithm using the MapReduce programming model, and introduce the motivating application and data set. In Section Results and discussion we discuss an imaging genetics study of Alzheimer's disease, and illustrate how the PaRFR algorithm detects several genes that have been previously reported in the literature. We also illustrate how PaRFR can be used to estimate a measure of genetic proximity, and investigate the correlation between genetic and phenotypic diversity, as well as the cumulative effects of multiple mutations on the severity of the disease. We conclude in Section Conclusions by providing an overview of alternative parallel RF algorithms and some remarks on further work.

## Methods

### Random Forest regression

We call  D the data set comprising *N *unrelated individuals or samples genotyped at *P *biallelic markers. For each individual, the markers are arranged in a data vector ***x****_i _*= (*x*_*i*1_, *x_i_*_2_, ..., *x_iP _*), for *i *= 1, ..., *N*. Depending on the coding scheme, different genetic models can be applied. For instance, assuming an additive genetic model, each *x_ij _*represents the count of minor alleles recorded at the *j_th _*locus--homozygote of minor allele is 2, heterozygote is 1 and homozygote of major allele is 0. The associated quantitative trait for each subject is assumed to be a *Q*-dimensional real-valued vector which we denote as ***y***_*i *_= (*y*_*i*1_, *y*_*i*2_, ..., *y*_*iQ*_), with *i *= 1, ..., *N*. In imaging genetics study designs, for instance, it is common that the sample size *N *is much smaller than min{*P*, *Q*}.

The RF algorithm builds an ensemble of regression trees, each one independently learned on a boot-strapped version of  D. The required number of trees in the forest, Ntree, is a user-defined parameter. The training data set for each tree is obtained by randomly sampling *N *subjects from  D with replacement. The tree building process is accomplished by introducing a second layer of randomness and involves selecting a random subset of Mtry candidate SNPs at each node, among the *P *available SNPs, in order to reduce the correlation among trees. In each tree, the best split at a node is determined by evaluating a split function for each value of a candidate SNP, and then selecting the SNP that maximises this function (see also Equation 2). To reduce bias, the trees are grown to a maximum depth with no pruning or otherwise until a minimum sample size has been reached; by default, we set this value to 5 for univariate trait and 20 for multivariate traits. We only consider binary trees, although in principle multi-way splits could also be accommodated with minor changes.

For each tree, all the subjects in  D that do not become part of the bootstrap sample used for training are collected together to form an *out-of-bag *(OOB) sample, which is used as a testing set. Approximately 63.2% of the subjects in  D are utilised as training data, while the remaining subjects are OOB samples. Each OOB sample is used to obtain an estimate for the prediction error (PE) for its tree and these estimates are then averaged across all trees to provide an overall estimate [22].

Although RF is deemed to be relatively insensitive to the choice of Ntree and Mtry, in practice, for large-scale GWAS involving a massive number of predictors, and possibly multivariate responses, these parameters must be tuned to achieve an optimal predictive performance and increase the statistical power of the algorithm to detect the true causative SNPs. As the number of trees in the forest increases, the OOB error rate is expected to converge to a theoretical prediction error according to the law of large numbers [22]. It is therefore important to select a sufficiently large number of trees to guarantee optimal performance and stable ranking.

### Split functions for multivariate traits

The node splitting rule determines how each tree in the forest is built, and depends on the particular predictive task at hand. For each node *j*, two operations are performed: (a) every allowable split on each SNP is examined; (b) the best of these split is selected, and the left and right daughter nodes are created. The initial node is the root node, which contains the entire data set  D, and the two operations above are then applied repeatedly to each daughter node until no more splits can be obtained. During this process the value of a split function *ϕ*(*j*) is computed for every split at node *j*. In regression tasks with both univariate and multivariate responses, sum-of-squares functions are commonly used [23]. In what follows, we let D(j) denote the subset of samples associated with node *j*, and $M_{j}$ the set of Mtry candidate SNPs that are available to split node *j*. Furthermore, the mean response vector observed in D(j) is denoted ȳ(j). With this notation in place, the total sum of squares at node *j *is

(1) $$SS\left(j\right)=\underset{i\in D\left(j\right)}{\sum }{\left(y_{i}-ȳ\left(j\right)\right)}^{T}V^{-1}\left(\Theta ,j\right)\;\left(y_{i}-ȳ\left(j\right)\right)$$

where V(Θ,j) is the *Q *× *Q *covariance matrix estimated from D(j), and depends on an unknown parameter vector Θ. A fully parametrized covariance matrix requires *Q*(*Q *+ 1)/2 parameters. With low sample sizes, a more parsimonious model is generally preferable so that Θ. has only a few unknown parameters. It is standard procedure to set the covariance matrices at the daughter nodes equal to the estimated covariance matrix at the parent node, in order to guarantee that the split function remains positive [23]. This procedure has the additional benefit of reducing the number of computations. We call *Standard RF *the algorithm that uses the splitting criterion (1).

When a SNP is selected as a candidate to split node *j *into two daughter nodes, the total sum of squares computed at the left and right daughter nodes are *SS*(*j*)*_l _*and *SS*(*j*)*_r_*, respectively. A suitable function in this case measures the reduction in the sum of squares due to the split, and is given by

(2) $$\phi \left(j\right)=SS\left(j\right)-SS{\left(j\right)}_{r}-SS{\left(j\right)}_{l}.$$

Every candidate SNP is tested to split the node, and the one with the highest *ϕ*(*j*) is selected. Once the best split has been found, the daughter nodes become new parent nodes and the covariance matrices are estimated again.

Parsimonious parametrisation of the covariance matrices are required in order to keep the computational burden low. In high-dimensional settings, and especially when *N *is much smaller than *Q*, it is commonplace to assume that the covariance matrices are diagonal [15,24]. For instance, typical whole-brain imaging genetics studies may involve a few hundred thousands brain-wide measurements while the sample remain in the order of a few hundreds [19]. In this situation, the total sum of squares of Eq. (1) can be alternatively expressed in terms of all *N *× *N *squared inter-point Euclidean distances between all *N *samples [25]. By rewriting Eq. (1) in an equivalent form, when V(Θ,j)= diag(1,…,1),

(3) $$SS\left(j\right)=\frac{1}{2N\left(j\right)}\underset{i\in D\left(j\right)}{\sum }\underset{l\in D\left(j\right)}{\sum }d_{E}^{2}\left(y_{i},y_{l}\right),$$

where *N*(*j*) indicates the sample size at node *j*. This strategy provides an equivalent but computationally more efficient way of evaluating the split function of Eq. (2). The evaluation of each *SS*(*j*) term has a cost complexity of O(*N*(*j*)^2^) instead of *N *(*j*) × *Q*. We call *Distance-based RF *the algorithm that uses the splitting criterion (3).

### Measure of variable importance for SNP ranking

One of the attractive features of RF for GWAS consists in its ability to perform SNP ranking by computing a measure of variable importance [5]. A commonly used and computationally simple procedure for SNP ranking consists in monitoring the value of the split function *ϕ*(*j*) every time a particular SNP has been selected, in each tree. This score, which we will refer to as the information gain importance score, usually produces ranking that are comparable with other variable importance measures, including those that rely on computationally intensive permutation procedures [22]. In the context of genetics studies, SNPs with the highest importance score are preferred candidates for further exploration. In the literature, this approach has been successfully used as a prescreening step to prioritise predictive SNPs [26].

### Hadoop implementation

RF implementations generally build trees sequentially. However, a sequential approach is highly inefficient, especially when each tree involves a large number of SNPs, and many trees are needed in order to obtain reliable measures of SNP importance and estimates of prediction error. RF can be easily parallelised because all trees are independently learned from randomised versions of the data. We describe here a parallel version of the RF regression algorithm that we have implemented using the MapReduce programming model for deployment on large Hadoop clusters. Broadly speaking, the approach consists in letting each node in the cluster build a certain number of trees in the forest, and then letting the system collect and aggregate the partial results from all trees in the ensemble, in an automated and fault-tolerant fashion.

**Figure 1:**
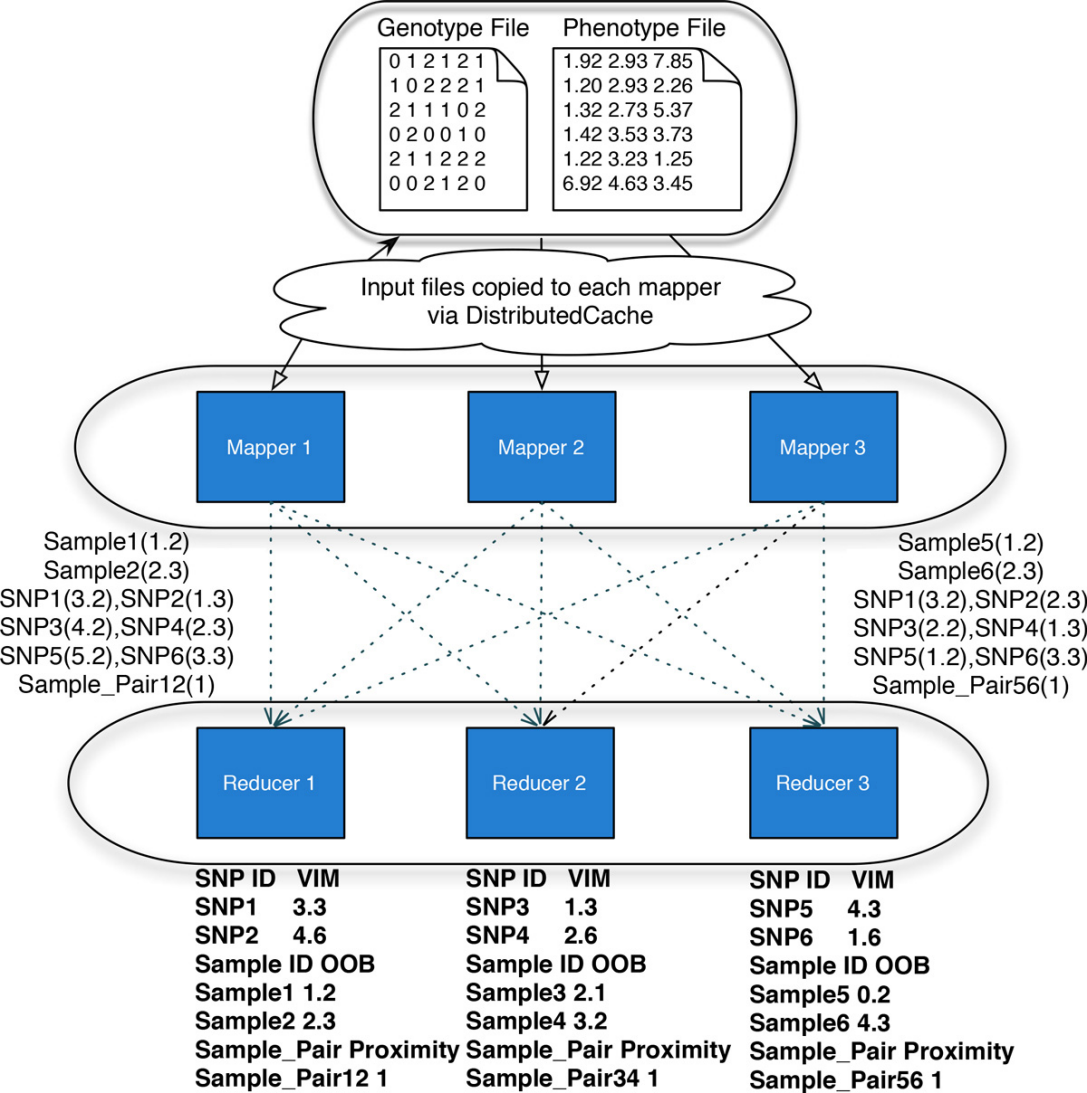
**PaRFR design**. An illustration of the RF algorithm implemented according to the MapReduce model. In this example there are 6 SNPs observed on 6 samples, and the analysis is carried out using 3 mappers and 3 reducers. The RF parameters here are set to Ntree = 3 and Mtry = 3.

The MapReduce model involves three phases: the *map *phase, the *shuffle *phase and the *reduce *phase. Each one of the *map *and *reduce *phase has key-value pairs as input and output. The *shuffle *phase shuffles the output of *map *phase to the input of *reduce *phase evenly using the MapReduce library. The *map *phase runs a user-defined *mapper *function on a set of key-value pairs [*k_j _*, *v_j _*] taken as input, and generates a set of intermediate key-value pairs. In the map phase of our application, each input key corresponds to a unique tree ID and value is NULL since we load the full data set to build trees. A user-defined number of mappers, nmap, are executed whereby each mapper function learns one or more decision trees from bootstrapped versions of the data set. The output of the map phase consists of three types of information: (1) Sample identifier (key) and predictive value, which is then used to estimate the OOB error rate at the reduce phase; (2) SNP identifier (key) and the decrease in sum-of-squares (value), which is used to obtain the SNP importance scores at the reduce phase; (3) Sample pair identifier (key) and its proximity (value), which is used to produce the final proximity matrix extracted from RF. All these outputs from mappers are sorted, shuffled, and copied to reducers by Hadoop. An illustration of this initial process is given in Figure 1. The Hadoop job initially distributes the data set to each map task using a *DistributedCache *mechanism, which copies the read-only files on to the slave nodes before launching a job. In our implementation, each map task loads the full version of the data set, which is then bootstrapped and used to learn each tree. In this illustration, Ntree = 3, and each one of the three mappers builds one tree. When the number of required trees is larger than the total number of mappers, each mapper builds more than one tree.

The output from all the mapper functions, consisting of key-value pairs, is then sorted, shuffled and copied to the *reduce *tasks, which receive their input in the form of [*k_j _*, [*v*_*j*__1_, *v*_*j*__2_, ...,]] pairs, where the first element can be SNP or sample identifier and the second element is a list of values associated with that SNP or sample. The reduce tasks run a user-defined reducer function, and generate an output again in the form of key-value pairs to be saved on file. The reduce tasks compute information gain importance score by summing up all the *ϕ*(*j*) evaluations obtained by the individual trees in the map phase. Again, the computations are equally distributed across reducers. For instance, in the bottom part of Figure 1, each reducer generates a partial list of key-value pairs containing SNP id and its information gain importance score. These lists are eventually saved on file and eventually they are joined to obtain the final output.

This parallel RF regression algorithm has been implemented in Java. It can be run in standalone, pseudo-distributed, and fully distributed mode. The current version, which supports biallelic markers, gives users the options to calculate OOB, variable importance scores, and the sample proximity matrix. Both standard and distance-based splitting criteria are available, and the Euclidean distance featuring in (3) could be easily replaced by other distances. For the standard RF version, currently both the Euclidean and Mahalanobis distances are available. An installation and user guide is available from the web site.

### The Alzheimer's Disease Neuroimaging Initiative cohort

This work was motivated by experimental data produced by the Alzheimer's Disease Neuroimaging Initiative (ADNI). Alzheimer's Disease (AD) is a moderate to highly heritable condition, and a growing list of genetic variants have been associated with greater susceptibility to develop early- and late-onset AD [27]. We have obtained genotypes for 253 unrelated subjects comprising 99 AD patients and 154 elderly healthy controls (CN). Genotyping was performed using the Human610-Quad Bead-Chip, which includes 620, 901 SNPs [28]. Subjects are unrelated, and all of European ancestry, and passed screening for evidence of population stratification using the procedure described in [19]. For this study, we include only autosomal SNPs, and additionally exclude SNPs with a genotyping rate <95%, a Hardy-Weinberg equilibrium p-value < 5 × 10^7^, and a minor allele frequency < 0.1. Missing genotypes were imputed as in [29].

For each subject in this study, longitudinal brain scans at 6, 12 and 24 months after the initial screening were available. Our multivariate quantitative trait provides a measure of structural change observed in the brain relative to baseline over the three time points. More specifically, each individual phenotype vector ***y****_i _*consists of 148, 023 slope coefficients, one for each voxel, quantifying the temporal rate of linear brain tissue loss over time, and therefore providing a localised imaging signature of the disease. A more detailed description of the preprocessing steps and the procedure used to extract this imaging signatures can be found in [30].

## Results and discussion

### Performance and scalability of PaRFR

Monte Carlo simulation studies were designed to compare the performance of our PaRFR software against other implementations of RF regression. In order to reduce the computational burden incurred in extensive and repeated simulations, we used a multivariate phenotype consisting of 100 voxels that were randomly selected from the 148, 023 available, and a set of 1, 000 SNPs randomly selected from the 434, 271 available markers. Using the 100 observed voxels, we estimated the phenotype sample covariance matrix, $\hat{V}$ Each artificial dataset consisted of the 253 samples for which the multivariate phenotype vector ***y****_i _*was generated by randomly drawing from a multivariate normal distribution with covariance matrix $\hat{V}$ and such that the genetic effects explain approximately 8% of phenotypic variability. Genetic effects were induced using an additive model involving 5 randomly selected causative SNPs with minimum allele frequency (MAF) 0.2, analogously to the simulation procedure described in [29]. The use of real data is important as it preserves the original patterns of linkage disequilibrium observed in the real dataset.

**Figure 2:**
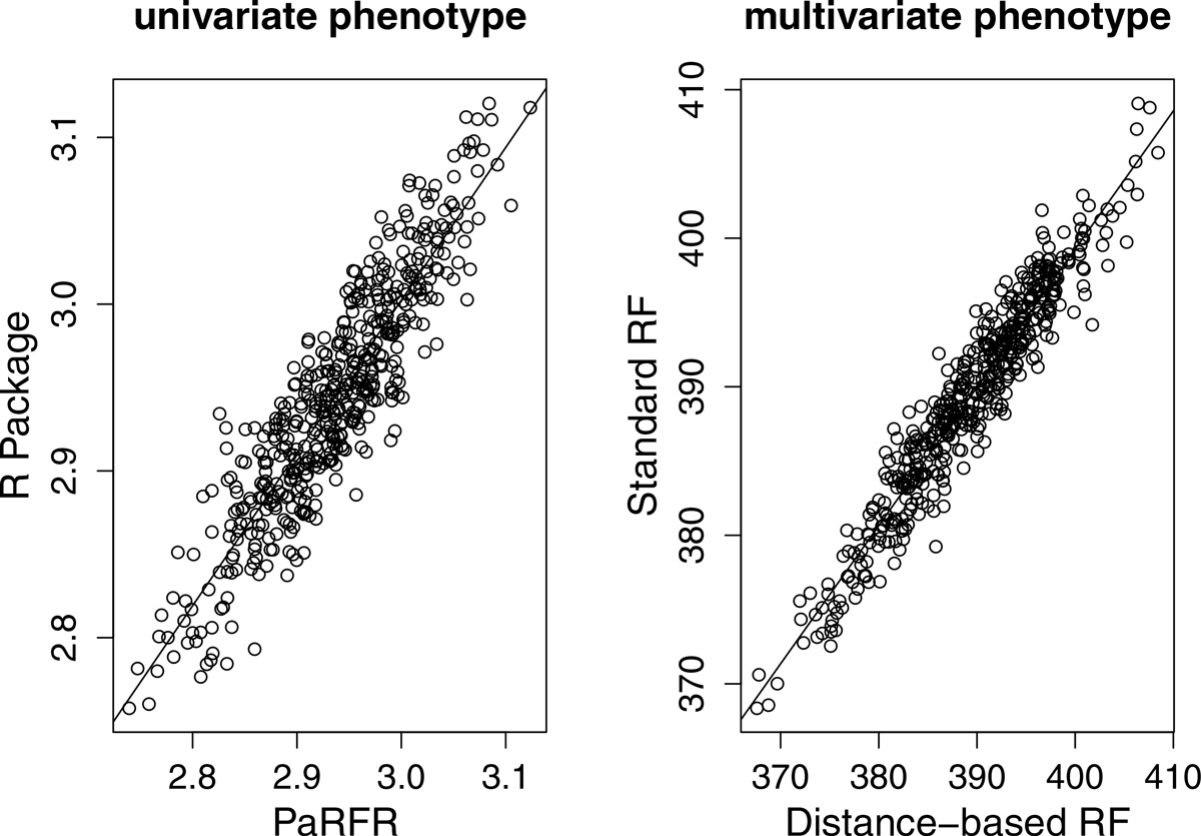
**The OOB error comparison**. Left: OOB error comparison with the *randomForest *implementation; Right: OOB comparison between the two multivariate node splitting criteria. In each case, we use 500 simulated datasets.

We first report on simulation experiments aimed to test and validate our RF implementation. We compare the OOB error obtained by PaRFR against the analogous OOB error obtained by using a publicly available implementation of RF in R package *randomForest*. Since the R implementation only handles univariate responses, for this specific evaluation we take the average across phenotypes, ȳ, as response. As can be seen in Figure 2 (left), the linear correlation coefficient between the two OOB errors is 0.91603 (p-value<0.001). Some discrepancy is expected due to the Monte Carlo error made during the tree building process. Secondly, we compare the two different node splitting criteria introduced in Section using the 100-dimensional artificial phenotypes. Figure 2 (right) shows that the two criteria produce highly correlated OOB errors, with a correlation coefficient of 0.93055 (pvalue<0.001). As before, the small difference in performance is explained by the randomness involved during the tree building process. In both cases, we report on average values obtained from 500 simulated data sets.

To assess the speedups that can be obtained by our implementation using the distance-based splitting function, we simulate a large dataset containing 1, 000 independent samples, 1 million SNPs and a 10, 000-dimensional phenotype. This analysis was run on a private cluster with 20 nodes. Each node had 24 GB memory and 16 processors with Intel(R) Xeon(R) CPU 2.27 GHz. We configured each *map *and *reduce *task to have 800 MB memory, and the whole cluster capacity to run 400 *map *tasks and 100 *reduce *task.

**Figure 3:**
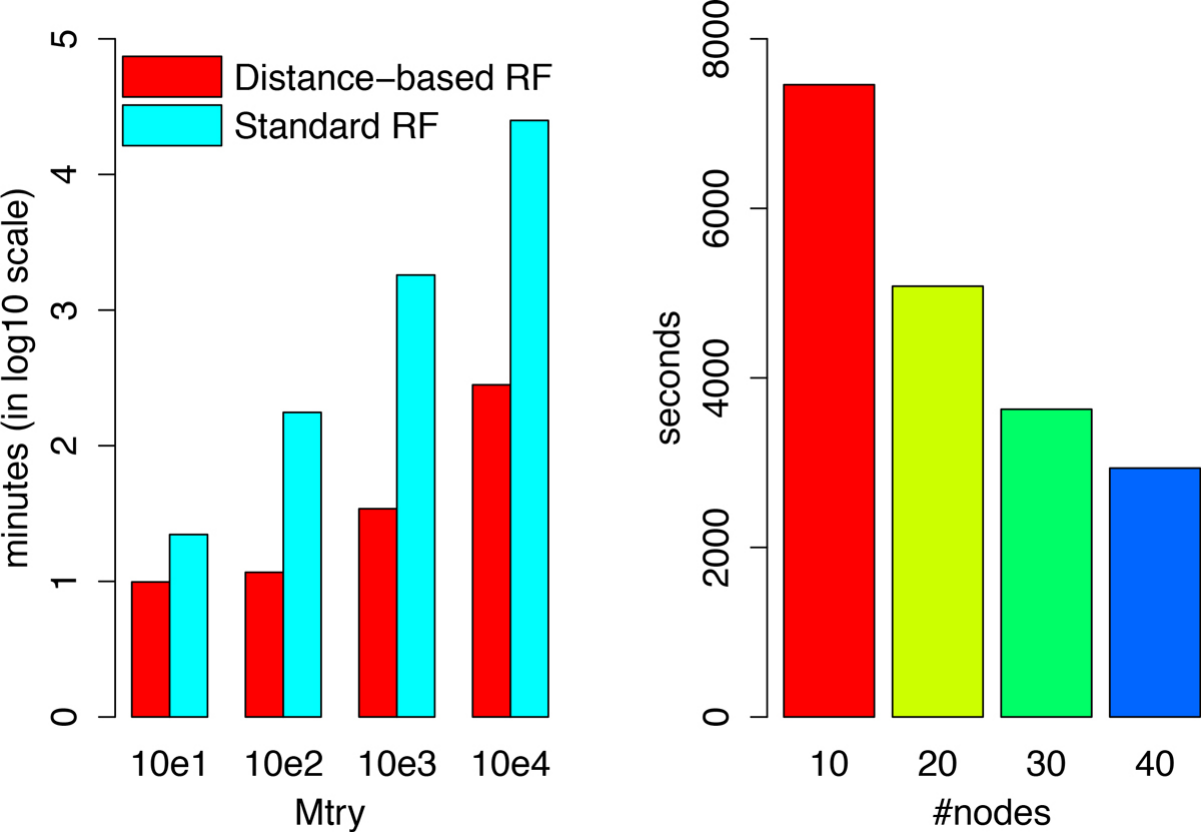
**The runing time comparison**. Left:runing time comparison using two different RF implementations for different Mtry; Right: the scalability test of Distance-based RF in local cluster.

We then compare the running time for different values of the Mtry parameter, ranging from 10 to 10, 000. Figure 3 shows the running time of the two methods on a log scale. When Mtry = 10, the distance-based RF is only 2 times faster than the Standard RF because of the initial time required to launch the cluster and load the data. As the value of Mtry increases, we observe at least a 10-fold speedup, indicating that significant running time savings can be obtained by using Distance-based RF for high-dimensional phenotypes.

To test the scalability of our implementation as the number of available nodes increases, we analyze the same size dataset with parameters Mtry = 1,000 and Ntree = 2,400. This analysis was run on our local cluster with 10, 20, 30 and 40 nodes, respectively. Figure 3 (right) shows that the running time decreases as more nodes are added and that, as expected, rough linear speedup is observed as the number of nodes increases. For instance, doubling the number of nodes from 20 to 40 yields a 40% reduction in running time.

### A GWA study of Alzheimer's disease

#### SNP ranking

In order to illustrate the benefits of the proposed PaRFR algorithm, we carried out the analysis of the ADNI data set. A handful of genetic variants with very large effect size are known to be related to AD, and we aimed at detecting those genes despite the relatively low sample size as a demonstration that PaRFR provides a useful toolbox for imaging genetic studies. Only the observed quantitative traits, and not the actual clinical status, were used by the algorithm. It is widely believed that such endophenotypes carry more signal than the cruder case-control status thus requiring much smaller sample sizes.

**Figure 4:**
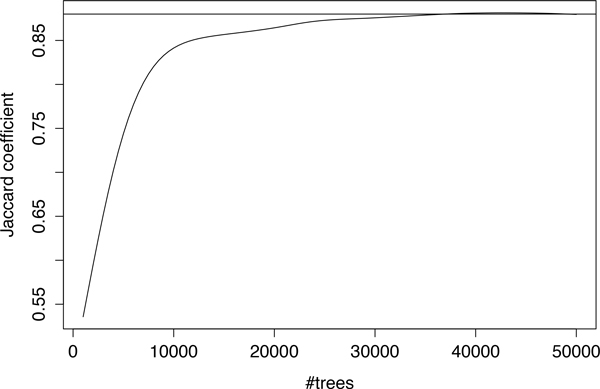
**Jaccard coefficient plot**. The Jaccard coefficient plot for the agreement of top 5,000 ranked SNPs with more trees added, the horizontal line is Jaccard coefficient = 0.88.

The analysis was run on our local cluster using 20 nodes. Different Mtry and Ntree parameter values were initially tested to determine how sensitive the results were to changes in these values, and in order to identify optimal values for which a stable SNP ranking can be produced. In particular, we explored various combinations of 3 different Mtry values and Ntree values varying from 10 up to 50, 000. The performance of PaRFR was monitored as more trees were added to the RF, in blocks of 500; once an additional block of trees was added to the ensemble, a measure of SNP ranking agreement with the previous ranking, the Jaccard coefficient, was calculated. Figure 4 shows how the Jaccard coefficient varies as more trees are added to the forest; this result indicates that a stable ranking is obtained when more than 40, 000 trees have been added. Based on this results, we settled for Ntree = 50,000 and used an optimised value of Mtry = 72, 379 as these parameters provided a final stable ranking. The total running time was 60 hours on a 20-node cluster, and the final RF ranked 352, 968 SNPs. Since the process of inferring an individual tree took approximately 20 minutes, we estimate that a sequential implementation using the same machines would approximately take 700 days.

**Figure 5:**
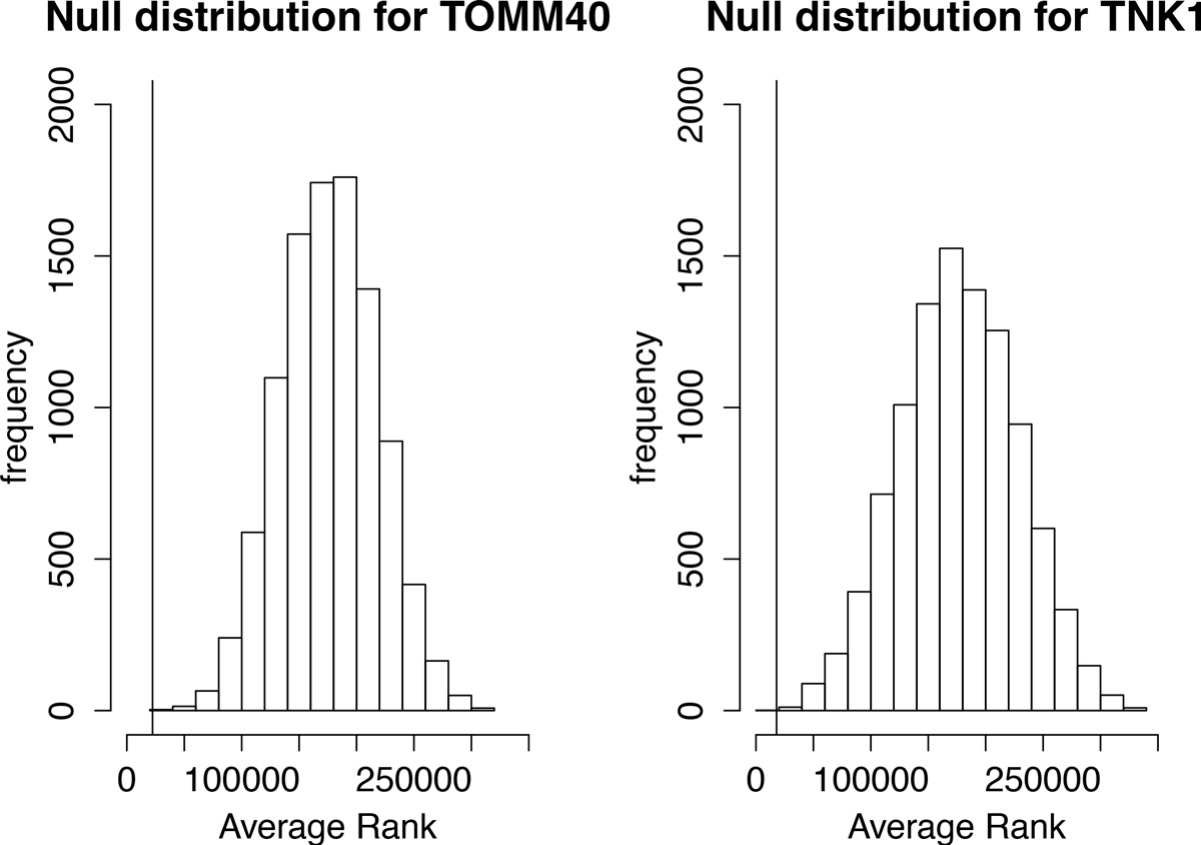
**The distribution of null ranks**. The Null distribution obtained by permuting 10,000 times the rank of SNPs harboured by the top 2 genes.

In Table 1 we report the top 10 ranked SNPs, using the information gain importance score. Any gene found to be within 10*k *bases of any of top 10 SNPs was considered mapped and, according to this criterion, we found 12 mapped genes. Several genes that have been reported to be linked to increased AD susceptibility are found in this list, including APOE4, TOMM40, PICALM and PVRL2 [20,27,28]. To further validate the relevance of the SNP rankings produced by PaRFR, we carried out a non-parametric analysis using permutation testing. First, we focused on the 47 well-known AD-linked genes reviewed by Saykin et al. [28], of which only 40 mapped to at least one SNP that had been ranked by PaRFR in our dataset. We then assessed the null hypothesis that the observed ranking of these genes, as produced by PaRFR, could be explained by chance only. For each mapped AD-linked gene *g*, we first obtained an observed score, ${ŝ}_{g}$, by averaging the ranks of all SNPs that map onto that gene. Operating under the null hypothesis that the AD-genes are randomly ranked, we permuted the SNP ranks 10, 000 times, and computed an empirical null distribution of the average rank score, *s_g_*. A p-value was then computed using this null distribution. As an example, the null distribution of 2 important genes, TOMM40 and TNK1, is shown in Figure 5 along with the observed score (vertical lines). Besides APOE4 and TOMM40, which are also among the top 10 genes, we also found TNK1, NXPH1, ACE, and MYH13 to have statistically significant average ranks while controlling the false discovery rate at 10%.

**Table 1:** 10 top ranked genes

Mapped Gene(s)	Ranked SNP(s)
TOMM40/APOE/APOC1*	APOE4
PICALM*	rs7938033
PVRL2*	rs2075650
NTNG2	rs7862808
NTM	rs12293070
SLC12A1	rs6493311/rs1531916
MEF2D	rs1750304
CD109	rs9352023
UNC5B	rs10762435
DPYD	rs496179

ADNI: top 10 genes and corresponding SNPs as ranked by PaRFR. The starred genes have been known to be linked with AD.

To understand the functional annotations of our top 12 mapped genes we used Gene Ontology (GO). GO provides a controlled vocabulary for evaluating complex function. The annotation files and GO tree (ver. 1.2) files for Homo Sapiens were downloaded from http://www.geneontology.org (dated 23 April 2011). As GO uses UniProt IDs while our genes were annotated using ENSEMBL IDs, we used Biomart to map UniProtKB accessions to Ensembl Gene IDs. This allows proper cross-comparisons. The through-path rule was applied for annotations to each gene, i.e. for a given GO term *G *annotated to some gene *p*, all the ancestral terms of *G *is also applied to *p*. This step was necessary because GO only maps a gene and/or its corresponding protein to the most specific term, which corresponds to some branch on the GO graph. To reduce redundancy and dependencies amongst the reported functional terms, informative biological process terms filtering was performed from the GO OBO file [31]. Significance testing for each cluster was performed using the hypergeometric test with Bonferroni correction (p-value ≤ 0.05). With this additional information, we were able to compare the list of our top 12 genes against the list of 47 known AD-linked genes. The comparison was carried out by comparing their GO terms and their corresponding agreement rate. The two lists of GO terms are available in Additional Files 1 and 2. Each list was tested against our reference database using a hypergeometric test with Bonferroni correction at a 5% nominal level. Overall, 47 GO terms from the top 12 mapped genes in our list were found to be statistically significant, and 14 of them agree with the 39 GO terms that are significant from the list of 47 known AD-linked genes. Compatibly with prior reports in the literature, these results further strengthen our belief that the top ranked genes are associated with an increased risk of AD.

#### Linking genetic and phenotypic diversity

Our PaRFR algorithm also estimates pair-wise genetic proximities in the form of a symmetric proximity matrix *P *of size *N *× *N *without any additional computations. Each element *p_ij _*of this matrix indicates the genetic similarity between a pair of samples, which is obtained by considering the fraction of trees where the two samples appeared in the same leaf. A measure of genetic distance between any two samples is then simply obtained by taking *d_ij _*= 1 − *p_ij _*. Further insights into the heritability of AD can be gained by analysing such estimated genetic distances with regards to the observed phenotypic distances. Specifically, we explored whether there was an association between phenotypic diversity, obtained from the neuroimaging measurements, and the inferred genetic distances. When any of these distances is used for clustering samples, we would expect to be able to identify at least two well-separated clusters of points corresponding to two the clinical conditions represented in the ADNI cohort, i.e. AD patients and healthy controls, despite the fact that the clinical status was not used by the PaRFR algorithm. In order to verify this, we applied multidimensional scaling (MDS) with the objective to visualise both genetic and phenotypic distances on a two-dimensional Euclidean space.

**Figure 6:**
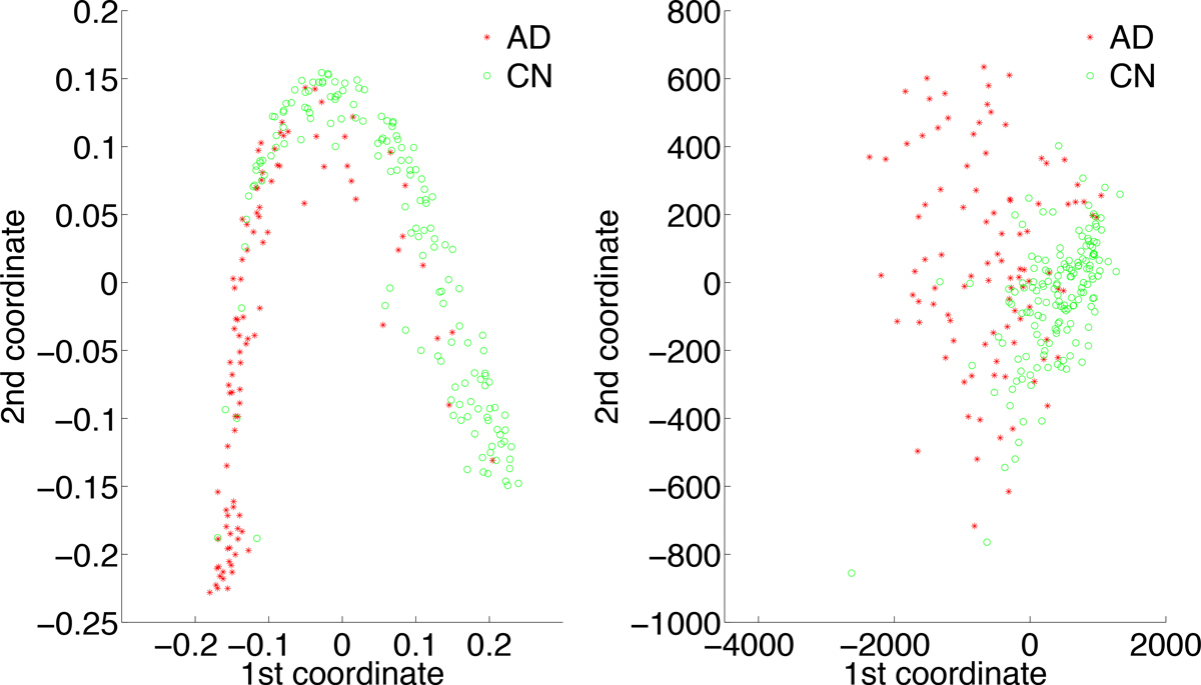
**Two-dimensional multidimensional scaling plots**. (a) 2D representation of the AD and CN samples obtained from the pair-wise genetic distances estimated by PaRFR; (b) 2D representation of the AD and CN samples obtained from the pair-wise Euclidean distances of the multivariate neuroimaging phenotypes (148, 023 voxels). Sample clustering can be seen in both plots.

**Figure 7:**
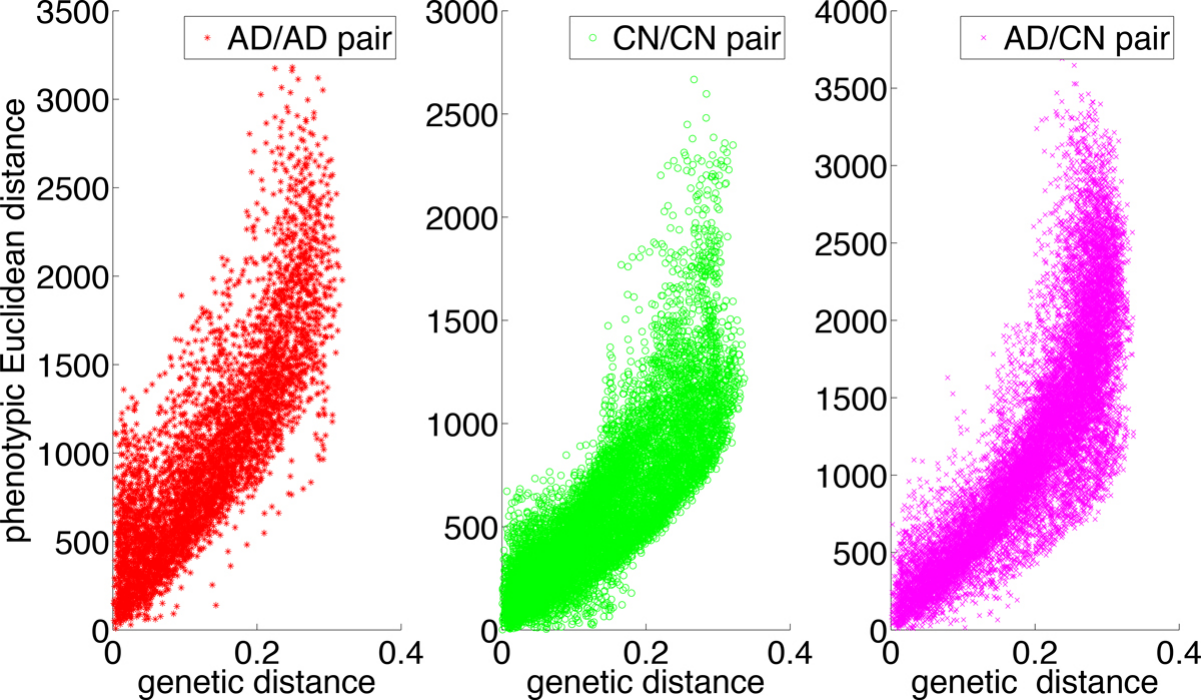
**The genetic distance and phenotypic distance correlation**. The three plots are the scatter plot of genetic Euclidean distance derived from Figure 6 left and phenotypic Euclidean distance derived from Figure 6 right for three types of sample pair. 4 outliers from CN groups are excluded.

Figure 6 shows the resulting MDS plots: (a) provides a 2D representation of the AD and CN samples obtained from the pair-wise genetic distances estimated by PaRFR, from which it can be noted a non-linear separation between AD and CN subjects; (b) provides a 2D representation of the AD and CN samples obtained from the pair-wise Euclidean distances applied to the neuroimaging signature consisting of 148, 023 voxels, which also shows a separation between the two phenotypically distinct groups, with higher variability characterising the AD samples. Remarkably, Figure 7 shows that the paired genetic and phenotypic distances are almost linearly associated; in these plots, the pair-wise comparisons are broken down by clinical group, i.e. AD-only samples, CN only samples, and combined samples. The linear correlation coefficients are 0.8248, 0.8245, and 0.7989, respectively, and indicate that the estimated genetic distance is predictive of phenotypic diversity. These results were further validated by performing a Mantel test of no association [32] between the paired genetic and phenotypic distance matrices in each one of the three cases; using 10, 000 permutations, all three tests were found to be statistically significant at the 0.01 nominal level.

#### Linking disease severity with mutation patterns

**Figure 8:**
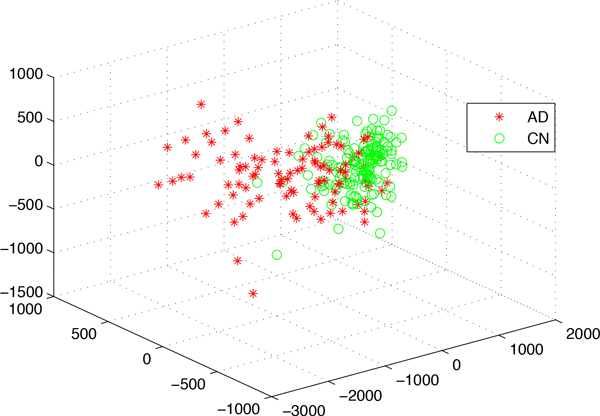
**The 3D MDS plot of the 148,023 voxels from 253 ADNI samples**. This plot is used to visualize the relative distance between different samples from high-dimensional space to 3 dimensions.

An alternative view of the phenotypic diversity characterising the samples is provided by the three-dimensional MDS plot of Figure 8. As in the two-dimensional representation of Figure 7, here it can be observed that the CN samples are more tightly clustered while the AD samples are more spread out, which is indicative of a large spectrum of disease progression. The variability in disease progression that can be observed here also reflects different degrees of disease severity, which in turn corresponds to different levels of cognitive impairment. It is therefore sensible to assume that the Euclidean distance between an AD-labelled point in this 3D space and a the centre of CN-labelled points provides a quantitative indicator of disease severity. For a complex disorder like AD, it is also plausible to assume that there is a large set of genetic markers, besides those with relatively large marginal effects, that jointly contribute to the disease and possibly determine its severity in each individual patient. Under the assumption that AD is the result of the cumulative effect of the dysfunction of multiple genes, we should then be able to observe an association between our indicator of disease severity and the number of mutations.

**Figure 9:**
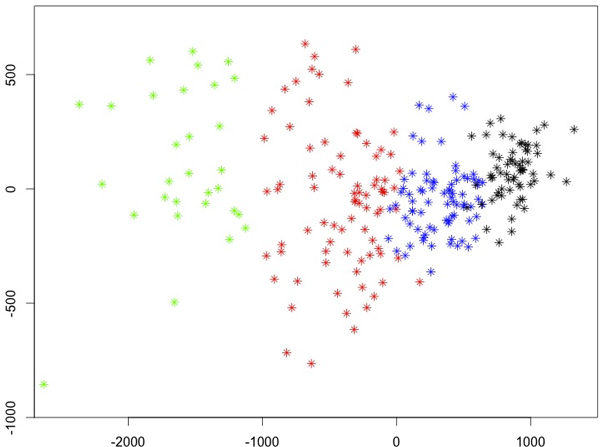
**The hierarchical clustering results of 253 ADNI samples**. The 2D MDS plot of the hierarchical clustering of 253 ADNI samples. The four clusters, from right to left, are referred to in the main text as C1, C2, C3 and C4.

**Figure 10:**
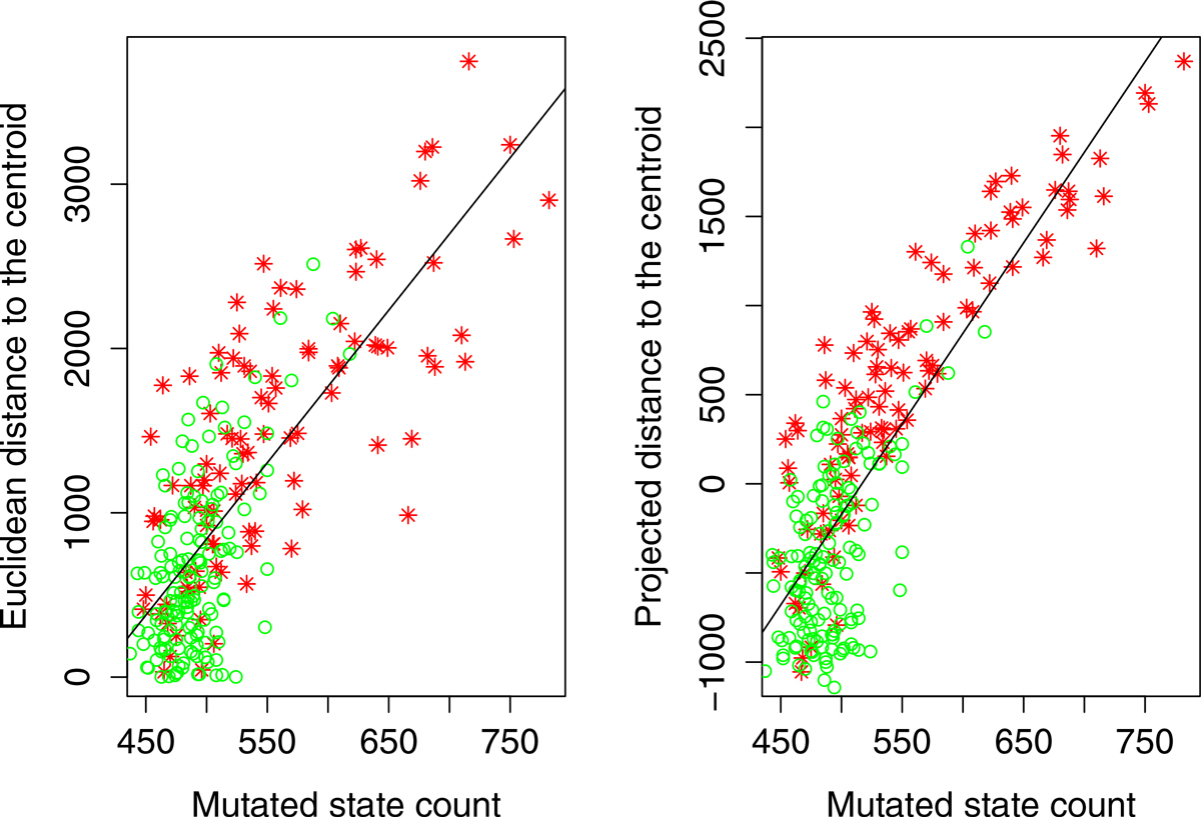
**The correlation between the severity of disease and the count of mutated state**. The correlation between the number mutated state and the distance to the healthy centroid (centroid of C1). Red stars are the AD samples and green circles are the CN samples. The fitted line are plotted because the beta coefficient of the line is statistically significant at p-value 0.05.

For this analysis we focused on the top 1, 000 SNPs ranked in decreasing order of importance by the PaRFR algorithm. For each one of these SNPs, we let the major allele be the *reference *state and the minor allele be the *mutated *state. In order to precisely define the severity of the disease for a given sample, we initially apply a hierarchical clustering algorithm to segment the samples into four non-overlapping clusters (from C1 to C4) as shown in Figure 9. The proportions of AD samples in these clusters are 11%, 16%, 63%, and 93%, respectively. The first notion of disease severity is defined as the Euclidean distance between an AD sample point and the centroid of points in the C1 cluster, which is mostly populated by CN samples. The second notion of severity is defined as the projected distance -- on the axis of disease progression-- of an AD sample to the centroid of C1 samples. The axis of disease progression is defined as the line connecting the centroids of the two extreme clusters, i.e. C1 and C4. We expect AD patients that are further away from the C1 centroid tend to have more mutated states compared to those that are nearer to the C1 centroid. Figure 10 is obtained by plotting the correlation between the count of mutated states and each one of two severity indicators defined above. Both correlations are positive and statistically significant at the 5% nominal level thus providing support for our hypothesis. From the bottom left part of the two plots, the CN samples are tightly clustered and have a lower number of mutated states, which is also in consistent with our expectations.

**Figure 11:**
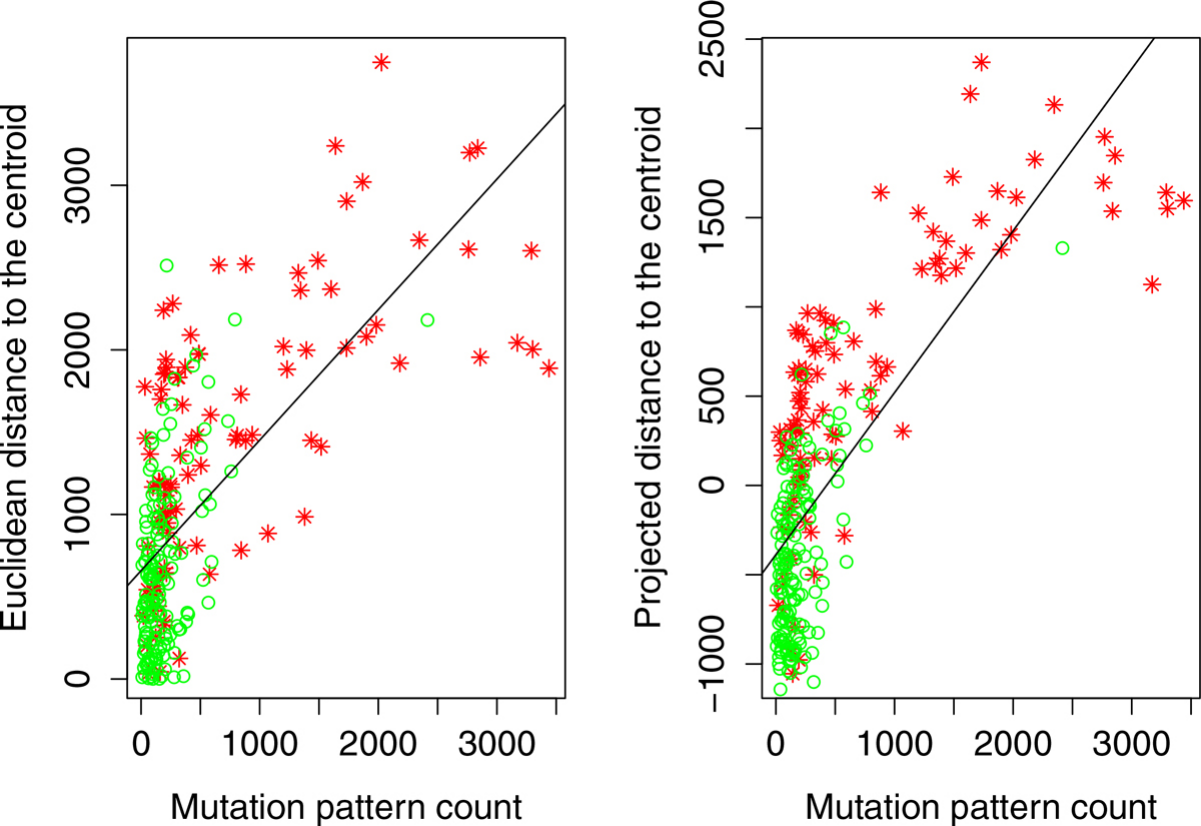
**The correlation between the severity of disease and the count of mutation patterns**. The correlation between the number mutation pattern and the distance to the healthy centroid (centroid of C1). Red stars are the AD samples and green circles are the CN samples. The fitted line are plotted because the beta coefficient of the line is statistically significant at p-value 0.05.

As a further refinement of this approach, we performed a second analysis in which we explored SNP/SNP combinations, or *mutation patterns*, in the genotypes containing mutated states that are frequently present in samples near the C4 centroid. For this analysis, we considered the top ranked 100 SNPs. All homozygous two-major-allele genotypes were removed in the data because disease-causing mutation patterns are expected to occur in the homozygous two-minor-allele genotypes and heterozygous genotypes. Only mutation patterns that were more frequently found in the C4 cluster were included in this analysis. Specifically, for each pattern we tested the null hypothesis that the proportions of that pattern in the C4 cluster and in the entire sample are equal, and only retained those patterns for which the null hypothesis was rejected at a 5% nominal level. We also required the frequency of each pattern to be at least 0.05 as a criterion for inclusion in the analysis. This procedure generated approximately 17, 000 mutation patterns. Figure 11 shows the correlation between the mutation pattern counts and our two measures of AD severity. Again, in both cases, the correlations are positive and statistically significant. As with the previous findings, this result supports our hypothesis that the number of mutation patterns carried by the AD samples is directly related to AD severity.

## Conclusions

In this paper we have introduced four contributions. First, we have proposed Random Forest regression with multivariate responses as a suitable approach to genetic association studies involving quantitative, and possibly high-dimensional, endophenotypes. This algorithm, which has been previously employed only in case-control studies, provides a natural framework for obtaining a ranked list of SNPs according to their predictive power. Second, we have proposed a parallel version of Random Forest regression, called PaRFR, which enable large-scale genetic association studies to be carried out in a computationally efficient way. The corresponding software, which has been made freely available, takes advantage of the Hadoop framework for distributed computing to build very large tree ensembles on large commodity clusters. We have also reported on extensive simulation results and comparisons of our implementation to a traditional (serial) implementation in terms of scalability and speed. Third, we have described an application of the proposed methodology to an imaging genetics study of Alzheimer's disease using quantitative traits extracted from brain scans by means of neuroimaging techniques. To the best of our knowledge, this is the first time that such a regression modelling methodology is used in imaging genetics. Lastly, we have demonstrated how the Random Forest regression algorithm can also be used to infer a measure of genetic similarity that is predictive of a quantitative phenotype, and have discussed this particular feature of the algorithm using the ADNI data set.

Other Hadoop-based implementations of the Random Forest regression algorithm currently exist, including COMET [33] and *Mahout *(http://mahout.apache.org). COMET is particularly suited for large data sets as it splits the input files into blocks of fixed size. However, this implementation only supports classification problems, and therefore is not suitable for handling quantitative traits, and is not open source. *Mahout *is an open-source machine learning library for large-scale data processing using Hadoop. Its latest version includes regression problems, but these are limited to univariate traits. Currently, other features are also missing in those implementations that are needed for performing genetic association studies as described in this paper, including mechanisms to generate SNP rankings and estimating sample proximities based on the genetic data. In future work we plan to improve further on the current implementation of PaRFR by building forests using partial data (or *splits*) generated by Hadoop for applications to much larger data sets. We also plan to build a multi-pass MapReduce implementation which can monitor changes in OOB error as the forest grows, and incorporate a sequential hypothesis testing procedure [38] to automatically determine the optimal number of trees.

Our proposed distance-based node splitting criterion can potentially allow for more general phenotypic distances to be used within the same framework. Alternative distances would be able to better capture the phenotypic variability observed in the populations, and may be appropriately selected depending on how the multivariate quantitative traits are measured. For instance, PaRFR can be used to detect SNPs that are highly predictive of variability in more complex neuroimaging phenotypes, such as brain connectivity networks, which are commonly represented as graphs (i.e. collections of nodes and links between pairs of nodes). Such networks describe the set of connections in the neural system, or connectome, in which nodes could be neurons or cortical areas, and edges could be axons or fibre tracts [39]. PaRFR only requires choosing a distance measure that captures certain aspects in which the brain graphs differ.

Although we were mostly motivated by applications in neuroimaging genetics, the algorithm proposed here has wider scope and can be used for any QTL mapping study involving a very large number of genetic markers and high-dimensional responses. For instance, there is recent interest in detecting genetic variability associated with facial shape, which can be quantified using 3D phenotypes obtained from statistical shape analysis [40]. Other multivariate traits arise, for instance, in eQTL mapping studies, where the phenotypes consist of gene expression abundances, or in longitudinal studies involving time series or repeated measurements [41].

## Competing interests

The authors declare that they have no competing interests.

## Authors' contributions

YW implemented the PaRFR algorithm, and co-wrote this manuscript. WG discussed and analysed the GWA study results to find overlapping informative GO terms. LW participated in discussion of the method, and proposed the approach based on frequent mutation patterns. GM proposed the idea for the study and RF algorithm, and guided the ADNI data analysis. Both LW and GM edited this manuscript. Data used in preparation of this article were obtained from the Alzheimer's Disease Neuroimaging Initiative (ADNI) database (http://adni.loni.ucla.edu). As such, the investigators within the ADNI contributed to the design and implementation of ADNI and/or provided data but did not participate in analysis or writing of this report. A complete listing of ADNI investigators can be found at http://adni.loni.ucla.edu/study-design/ongoing-investigations. All authors read and approved the final manuscript.

## Supplementary Material

Additional file 1 **GO terms mapped by 47 AD linked genes**. The file contain three columns, the first column is the id of GO terms, the second column is the name of GO terms and the third column is the p-value of hypergeometric test. Click here for file

Additional file 2 **GO terms mapped by 12 top-ranked genes ranked PaRFR**. The file contain three columns, the first column is the id of GO terms, the second column is the name of GO terms and the third column is the p-value of hypergeometric test. Click here for file

## Declarations

Publication of this article was funded by Singapore Agency for Science Technology & Research grant SERC 102 101 0030.

This article has been published as part of *BMC Bioinformatics *Volume 14 Supplement 16, 2013: Twelfth International Conference on Bioinformatics (InCoB2013): Bioinformatics. The full contents of the supplement are available online at http://www.biomedcentral.com/bmcbioinformatics/supplements/14/S16.
